# Neurosarcoidosis: When Altered Mental Status Is a Sign of Systemic Disease

**DOI:** 10.7759/cureus.101504

**Published:** 2026-01-14

**Authors:** Nidia Oliveira, Elisa Veigas, Tânia Batista, Luís Abreu, Marco Bousende

**Affiliations:** 1 Internal Medicine, Unidade Local de Saúde de Viseu Dão-Lafões, Viseu, PRT; 2 Neurology, Unidade Local de Saúde de Viseu Dão-Lafões, Viseu, PRT; 3 Neuroradiology, Unidade Local de Saúde de Viseu Dão-Lafões, Viseu, PRT

**Keywords:** corticosteroid therapy, granulomatous diseases, multidisciplinary management, neurological manifestations, neurosarcoidosis, rare clinical presentation

## Abstract

Sarcoidosis is a systemic granulomatous disease of unknown etiology, characterized by a heterogeneous clinical course and the potential to affect any organ. Neurological involvement is rare and often mimics other nervous system disorders, with diagnosis based on the correlation of clinical, radiological, and histological findings, and biopsy of a suspected lesion serving as the definitive diagnostic method.

We report the case of a 59-year-old woman, previously independent, who presented with progressive cognitive decline over 4-5 weeks, culminating in the inability to perform basic activities. Based on clinical, laboratory, and imaging findings, including magnetic resonance imaging and FDG-PET, a diagnosis of neurosarcoidosis (NS) was established, and oral corticosteroid therapy was initiated, resulting in significant clinical and imaging improvement. Although histological confirmation was not obtained, the integration of clinical and imaging findings provided consistent support for the diagnosis.

This case highlights the importance of early recognition of NS, particularly in subacute presentations with cognitive impairment.

## Introduction

Sarcoidosis is a systemic granulomatous condition of unknown etiology, histopathologically characterized by the formation of non-caseating granulomas that can affect multiple organs, with the lungs being the most commonly involved. The disease can occur in individuals of any age or ethnic group but is more frequent in young adults, with diagnosis typically established before the age of 50 [1-3].

Although respiratory symptoms are the most common, sarcoidosis can manifest in various organs, including the central nervous system, resulting in neurosarcoidosis (NS), a relatively rare form present in 5-10% of cases [4,5]. Neurological involvement is clinically relevant, as delays in diagnosis and treatment may lead to permanent neurological damage and long-term disability [4,5].

NS represents a diagnostic challenge due to the heterogeneity of its clinical presentation and its overlap with other neurological conditions [2]. The most frequent presentations include aseptic meningitis, encephalopathy, hydrocephalus, myelopathy, cranial neuropathy, intracranial mass lesions, vasculopathy, seizures, and hypothalamic-pituitary abnormalities [3,6].

The diagnosis of NS requires integration of clinical manifestations with laboratory findings, such as serum levels of angiotensin-converting enzyme (ACE) and calcium, neuroimaging studies, particularly brain magnetic resonance imaging, and, in selected cases, cerebrospinal fluid analysis via lumbar puncture [3,6].

According to the 2018 Neurosarcoidosis Consortium, NS can be classified into three categories: possible, probable, and definitive [7]. Possible NS is characterized by suggestive neurological symptoms in patients with previously diagnosed sarcoidosis, but without histological confirmation in the CNS. The diagnosis is based on the correlation of clinical, radiological, and laboratory findings [7]. When there are clinical or radiological findings of sarcoidosis in the CNS, such as leptomeningeal enhancement on MRI or PET-FDG, along with elevated biomarkers in the CSF, such as ACE and sIL-2R, the condition is classified as probable NS [7]. Definitive NS is confirmed by biopsy of the CNS or CSF analysis showing non-caseating granulomas typical of sarcoidosis, or when there is strong concordance between clinical, imaging, and laboratory findings, with significant clinical improvement following the initiation of corticosteroid treatment [7].

We report the case of a 59-year-old woman with progressive cognitive deterioration over 4-5 weeks. Laboratory investigations, including autoimmune markers, tumor markers, serologies, and ACE levels, revealed no abnormalities. F18-PET demonstrated mediastinal lymphadenopathy suggestive of active sarcoidosis, and brain magnetic resonance imaging showed leptomeningeal enhancement consistent with an inflammatory meningeal process, without nodular enhancement. Based on clinical, laboratory, and imaging findings, a diagnosis of NS was established, and oral corticosteroid therapy was initiated, resulting in significant clinical and radiological improvement.

This case highlights the importance of early diagnosis of NS, particularly in subacute cognitive presentations, in order to prevent the development of severe and disabling complications.

## Case presentation

We report the case of a 59-year-old woman, independent in her activities of daily living, with a medical history of arterial hypertension, type 2 diabetes mellitus, hyperthyroidism, and depression, who presented to the emergency department with a progressive alteration in mental status evolving over approximately four to five weeks. She initially exhibited confused and incoherent speech, which gradually progressed to markedly disorganized discourse focused on past events and deceased individuals, accompanied by loss of awareness of current reality and inability to perform basic daily tasks such as cooking or managing her medications. She denied fever, unintentional weight loss, night sweats, headaches, nausea, vomiting, diarrhea, cough, sputum production, dysuria, or other symptoms suggestive of an infectious source.

On evaluation, she was hemodynamically stable, with a blood pressure of 127/83 mmHg, heart rate of 79 bpm, afebrile, breathing comfortably on room air, and with an oxygen saturation of 98%. Cardiac and pulmonary auscultation and abdominal examination were unremarkable. Neurological examination revealed psychomotor slowing; she was alert and oriented to person but disoriented to time and place. Pupils were equal and reactive to light, visual fields were intact, and no nystagmus or facial asymmetry was observed. Muscle strength was preserved in all extremities, with symmetric osteotendinous reflexes. No abnormal movements or meningeal signs were present.

A non-contrast cranial computed tomography performed in the emergency department revealed no acute ischemic or hemorrhagic lesions. Initial laboratory evaluation, including complete blood count, renal and hepatic function tests, electrolytes, and inflammatory markers, was within normal limits (white cell count 5.57 x10^9/L (reference range: 3.90-10.20), neutrophil 3.7 x10^9/L (reference range: 42.00-77.00), and C-reactive protein 0.08mg/dL (reference range: 0.00-0.50)). Given the presentation of encephalopathy of unclear etiology, without identifiable metabolic or infectious triggers, a lumbar puncture was performed, which excluded CNS infection (negative viral panel, Gram stain and aerobic culture without microbial growth). The patient was admitted for clinical observation and further etiological investigation of the encephalopathy.

**Figure 1:**
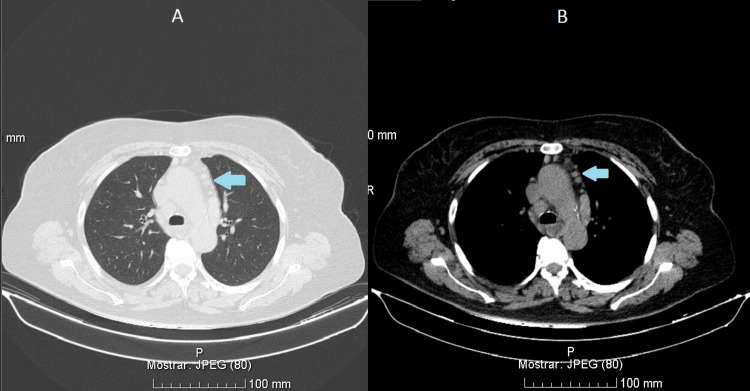
Chest computed tomography demonstrated a solitary, well-defined 7 × 5 mm pulmonary nodule in the left upper lobe, along with multiple anterior mediastinal lymphadenopathies (A: lung window; B: mediastinal window)

During hospitalization, additional diagnostic evaluation included chest computed tomography, which revealed a solitary, well-defined 7 × 5 mm pulmonary nodule in the left upper lobe, along with multiple anterior mediastinal lymphadenopathies (Figure 1).

F18-PET demonstrated hypermetabolic mediastinal lymphadenopathy suggestive of inflammatory nodal involvement compatible with active sarcoidosis, although other etiologies could not be excluded.

Endobronchial ultrasound-guided needle sampling of mediastinal lymph nodes yielded insufficient material for histopathological diagnosis. Bronchoscopy revealed no endobronchial abnormalities, and bronchial aspirates submitted for microbiological studies and flow cytometry were negative.

Electroencephalography showed global slowing of background activity, consistent with diffuse cerebral dysfunction and encephalopathy.

**Figure 2:**
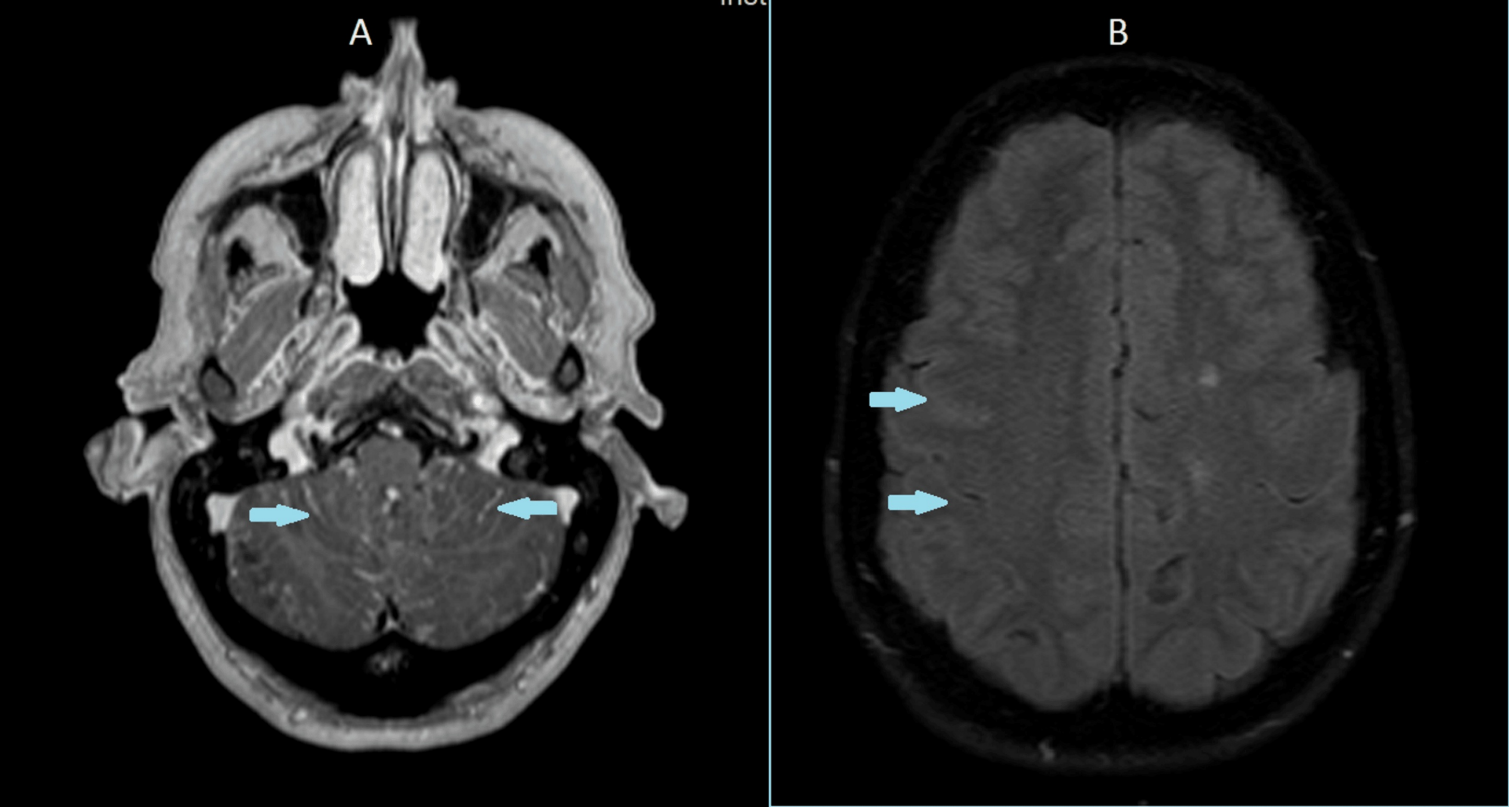
Contrast-enhanced brain magnetic resonance imaging demonstrated bilateral cortical and sulcal FLAIR hyperintensity with leptomeningeal enhancement involving the cerebral hemispheres, cerebellum, and brainstem, without nodular enhancement-findings compatible with an inflammatory or infectious meningeal process. A subtle periventricular hyperintensity suggestive of transependymal edema was also noted (A: Axial plane (posterior fossa); B: Axial plane (supratentorial/cerebral hemispheres)) FLAIR: Fluid-attenuated inversion recovery

Contrast-enhanced brain magnetic resonance imaging demonstrated bilateral cortical and sulcal fluid-attenuated inversion recovery (FLAIR) hyperintensity with leptomeningeal enhancement involving the cerebral hemispheres, cerebellum, and brainstem, without nodular enhancement, findings compatible with an inflammatory or infectious meningeal process. A subtle periventricular hyperintensity suggestive of transependymal edema was also noted (Figure 2). 

Laboratory investigations, including autoimmune markers, tumor markers, serologies, and ACE levels, revealed no abnormalities. A second lumbar puncture, including autoimmune and paraneoplastic studies of cerebrospinal fluid and serum, yielded negative results.

Based on the overall clinical, laboratory, and imaging findings, a diagnosis of NS was established, and the patient was started on oral corticosteroid therapy with prednisolone 60 mg/day (1 mg/kg/day). She demonstrated favorable clinical improvement and was discharged with follow-up in Internal Medicine, Neurology, and Pulmonology, with instructions for gradual tapering of corticosteroids.

**Figure 3:**
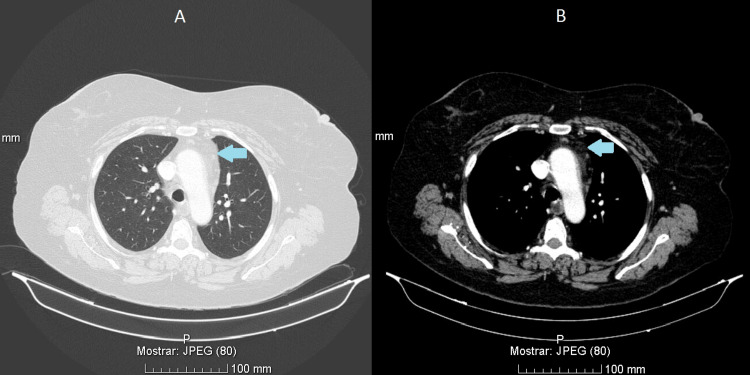
Chest computed tomography showing a marked reduction in the size of mediastinal and hilar lymph nodes, currently measuring less than 1 cm, with no criteria for lymphadenopathy (A: lung window; B: mediastinal window)

At follow-up, the patient exhibited significant clinical recovery, with restored orientation, coherent speech, and full independence in activities of daily living, including caring for a grandchild. Follow-up chest computed tomography showed marked reduction in mediastinal and hilar lymphadenopathies, with stability of the previously identified pulmonary nodule (Figure 3).

**Figure 4:**
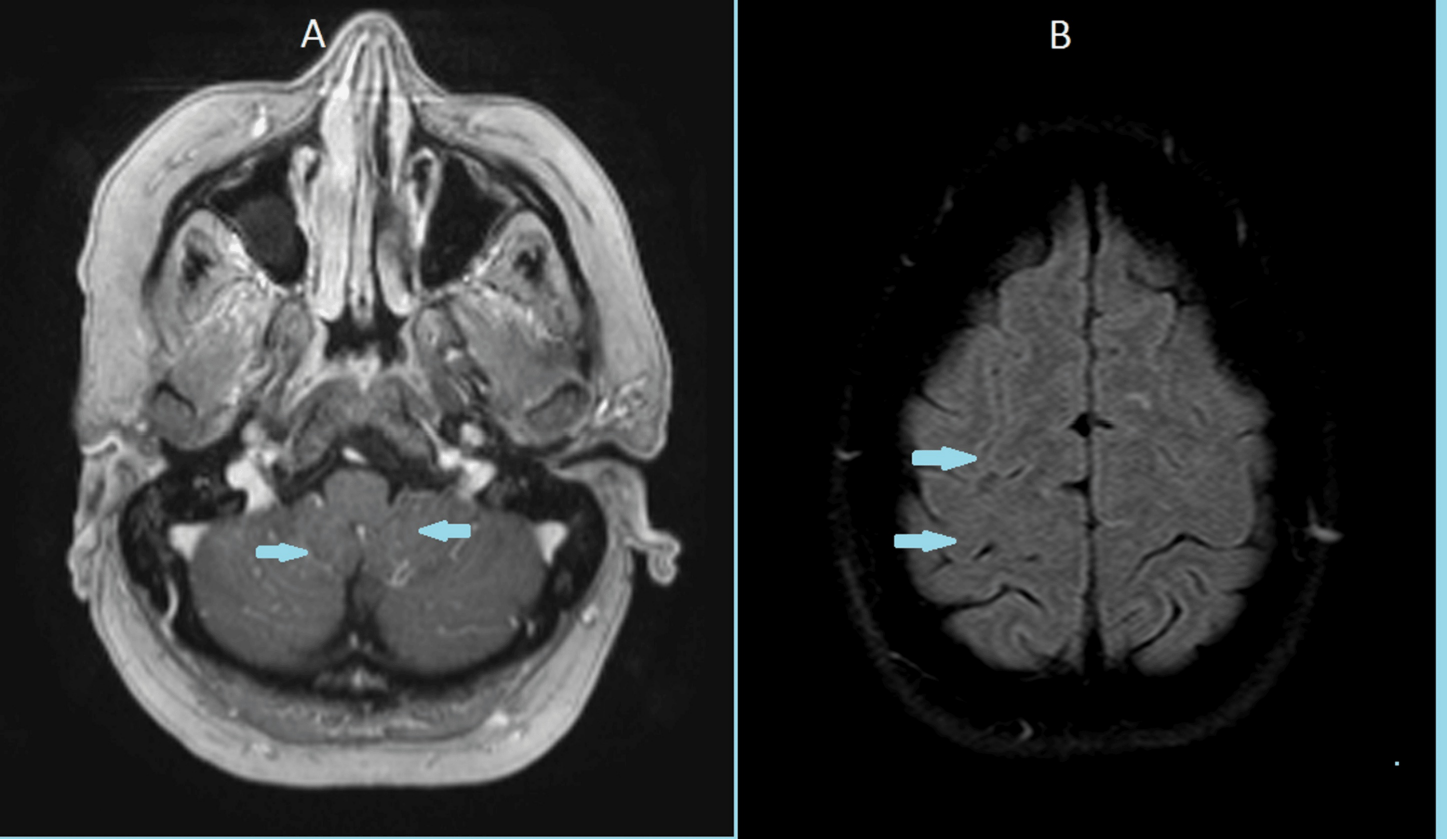
Brain magnetic resonance imaging demonstrates persistent meningeal enhancement in the posterior fossa, with minimal (nearly absent) enhancement in the cerebral hemispheres. Cortical and sulcal FLAIR hyperintensities have significantly decreased compared with the previous study, indicating radiological improvement (A: Axial plane (posterior fossa); B: Axial plane (supratentorial/cerebral hemispheres)) FLAIR: Fluid-attenuated inversion recovery

Repeat brain MRI demonstrated near-complete resolution of leptomeningeal enhancement, with only mild residual reinforcement in the posterior fossa, reflecting radiological improvement compared with the prior examination (Figure 4).

The patient remains under regular follow-up in the aforementioned specialties, currently using azathioprine 50 mg/day and prednisolone 5 mg/day, clinically stable and without recurrence of symptoms, on a regimen of gradual reduction of the corticosteroid dose.

## Discussion

NS is a rare and diagnostically challenging entity, characterized by a wide spectrum of clinical manifestations and the absence of a specific confirmatory test [8]. It may involve any region of the nervous system and frequently mimics other neurological or systemic disorders, further complicating early recognition [4,5,8]. In the present case, a previously independent patient developed a progressive alteration in mental status, with confused and disorganized speech focused on past events and deceased individuals, accompanied by loss of awareness of current reality and inability to perform basic daily tasks.

The diagnosis of NS relies on the integration of clinical suspicion, evidence of systemic involvement, and neuroimaging findings, with histological confirmation through biopsy demonstrating non-caseating granulomas [3,6,9]. One of the major challenges is the difficulty in obtaining neural tissue for histological and microbiological analysis, which makes MRI the preferred modality for evaluating the brain and spinal cord [8,9]. FDG-PET may serve as an important adjunct, providing information on metabolic activity, identifying occult disease sites, and aiding in treatment monitoring [8,9].

Although complementary tests such as blood work and CSF analysis can assist in excluding alternative etiologies, including inflammatory or infectious CNS processes, which were unremarkable in this case, imaging studies proved crucial for establishing the diagnosis. Chest computed tomography revealed a left pulmonary nodule with mediastinal lymphadenopathy, while FDG-PET demonstrated hypermetabolic mediastinal nodes consistent with active sarcoidosis. Cranial MRI showed gadolinium-enhancing meningeal involvement affecting the cerebral parenchyma, cerebellum, and brainstem, suggestive of an inflammatory process without nodular enhancement.

A multidisciplinary approach involving clinicians, radiologists, and pathologists is essential to achieve an accurate and timely diagnosis of NS [8]. First-line therapy consists of high-dose corticosteroids, as spontaneous remission is uncommon and ongoing inflammation may lead to permanent neurological deficits. Immunosuppressive agents, such as methotrexate, azathioprine, or mycophenolate mofetil, are reserved for patients with inadequate response to corticosteroids [8,9].

In the reported case, the patient showed significant clinical improvement following initiation of corticosteroid therapy, highlighting the effectiveness of this approach.

This case underscores the importance of considering NS in patients with atypical neurological manifestations and reinforces the need for thorough clinical evaluation, appropriate imaging studies, and histological confirmation whenever feasible.

## Conclusions

This case highlights the diagnostic challenges of subacute NS presenting with progressive cognitive impairment in the absence of systemic infectious signs. Diagnosis was established through clinical and radiological correlation, given the lack of specific laboratory markers. Early recognition and prompt corticosteroid therapy led to complete clinical recovery and marked radiological improvement. This report underscores the importance of timely diagnosis to prevent long-term neurological sequelae.
